# Reply to Drayman, “Observed High Coinfection Rates Seem To Be a Result of Overlapping Plaques”

**DOI:** 10.1128/mBio.01201-17

**Published:** 2017-08-08

**Authors:** Julie K. Pfeiffer

**Affiliations:** University of Texas Southwestern Medical School, Dallas, Texas, USA; Vanderbilt University Medical Center

**Keywords:** coinfection, mutagen, plaque, poliovirus

## REPLY

On behalf of my coauthors, I thank Dr. Drayman for his interest in our recent paper in which we examined whether plaques can contain more than one parental virus (1). We found that a small percentage of plaques (5 to 7%) contain multiple parental viruses, and we concluded that virion aggregation contributes to chimeric plaque formation. In a distinct flow cytometry-based assay that used a higher MOI and detection based on reporter protein production rather than plaque formation, we found that coinfection frequencies were detectable but relatively low compared with those observed in the plaque-based assay.

Dr. Drayman presents two explanations for the high frequency of observed chimeric plaque formation: (i) plaques are not products of coinfection, but instead overlapping plaques were picked and thus appear to be chimeric, and (ii) chimeric plaques are the products of coinfection, which provides a fitness advantage that increases the probability of successful plaque formation. Based on his simulations, Dr. Drayman proposes that plaques contain multiple parental viruses not because of coinfection but because of overlapping plaques.

Indeed, during our study we were aware of and concerned about the possibility that chimeric plaques were from overlapping plaques rather than bona fide coinfection. However, two results presented in the paper and a third result from our more recent study suggest that chimeric plaques are the products of bona fide coinfection and that coinfection provides a fitness advantage that aids infection.

First, we reasoned that if overlapping plaques were responsible for “chimeric” plaques, then plates with more plaques present should have more overlapping plaques and therefore have more “chimeric” plaques. In Fig. 1D, we showed that this is not the case: chimeric plaques were not overrepresented on plates with higher plaque numbers, indicating that cross-contamination or overlapping plaques were unlikely (1). Second, in Fig. 5, we showed that mutagenized viruses form plaques that have elevated coinfection frequencies (1). These mutagenized viruses generated only a small number of plaques per plate due to reduced specific infectivity, yet a relatively large number (17%) of plaques from these viruses were chimeric. Third, in our recent preprint (2), we demonstrate that bacteria can promote viral coinfection even when very few virions are present and, importantly, that this process facilitates viral genetic recombination and fitness restoration.

Overall, we think it likely that the majority of chimeric plaques are products of coinfection and that coinfection provides a fitness advantage that aids plaque formation. However, it is certainly possible that a subset of chimeric plaques may be due to overlapping plaques rather than coinfection, as Dr. Drayman suggests. But getting back to Dr. Drayman’s initial question—why is the apparent coinfection frequency much higher in the plaque-based assay than in the flow cytometry-based assay? We do not know the answer, but we hypothesize that plaque formation is much more difficult than expression of a reporter protein during a single cycle of infection in the flow cytometry assay and therefore that coinfection promotes productive plaque formation.
